# Global Interactomics Connect Nuclear Mitotic Apparatus Protein NUMA1 to Influenza Virus Maturation

**DOI:** 10.3390/v10120731

**Published:** 2018-12-19

**Authors:** Md Niaz Rahim, Ludger Klewes, Ali Zahedi-Amiri, Sabine Mai, Kevin M. Coombs

**Affiliations:** 1 Department of Medical Microbiology, Max Rady College of Medicine, University of Manitoba, Winnipeg, MB R3E 0J6, Canada; mdniaz.rahim@canada.ca (M.N.R.); zahediaa@myumanitoba.ca (A.Z.-A.); 2 Manitoba Centre for Proteomics & Systems Biology, Room 799, 715 McDermot Avenue, Winnipeg, MB R3E 3P4, Canada; 3 Department of Physiology and Pathophysiology, Max Rady College of Medicine, University of Manitoba, Winnipeg, MB R3E 0J6, Canada; Ludger.Klewes@umanitoba.ca (L.K.); Sabine.Mai@umanitoba.ca (S.M.); 4 Genomic Centre for Cancer Research and Diagnosis, Research Institute in Oncology and Hematology, ON6026-675 McDermot Avenue, Winnipeg, MB R3E 0V9, Canada; 5 Children’s Hospital Research Institute of Manitoba, Room 513, John Buhler Research Centre, 715 McDermot Avenue, Winnipeg, MB R3E 3P4, Canada

**Keywords:** Influenza A virus (IAV), monoclonal antibodies (mAbs), immunoprecipitation (IP), Western blotting, knockdown (KD), siRNA, Nuclear mitotic apparatus protein 1 (NUMA1), Viral replication

## Abstract

Influenza A virus (IAV) infections remain a major human health threat. IAV has enormous genetic plasticity and can rapidly escape virus-targeted anti-viral strategies. Thus, there is increasing interest to identify host proteins and processes the virus requires for replication and maturation. The IAV non-structural protein 1 (NS1) is a critical multifunctional protein that is expressed to high levels in infected cells. Host proteins that interact with NS1 may serve as ideal targets for attenuating IAV replication. We previously developed and characterized broadly cross-reactive anti-NS1 monoclonal antibodies. For the current study, we used these mAbs to co-immunoprecipitate native IAV NS1 and interacting host proteins; 183 proteins were consistently identified in this NS1 interactome study, 124 of which have not been previously reported. RNAi screens identified 11 NS1-interacting host factors as vital for IAV replication. Knocking down one of these, nuclear mitotic apparatus protein 1 (NUMA1), dramatically reduced IAV replication. IAV genomic transcription and translation were not inhibited but transport of viral structural proteins to the cell membrane was hindered during maturation steps in NUMA1 knockdown (KD) cells.

## 1. Introduction

Influenza A virus (IAV) remains a significant pathogen that causes substantial amounts of contagious respiratory disease in humans. Recent human infections with avian influenza viruses such as H5N1 and H7N9 subtypes emphasize the ongoing threat of this virus to cause future epidemics and pandemics. Two classes of anti-influenza drugs, viral M2 and NA inhibitors, are available; however, emergence of drug-resistant IAVs is becoming a serious concern [1,2,3,4]. In the future, influenza virus may lose sensitivity to all available drugs due to its genetic plasticity caused by the segmented nature of genomes and high mutation rate. Thus, there is a continued need to develop new concepts and drugs to overcome the problem of virus-targeted antiviral resistance.

All viruses are obligate intracellular parasites. Successful viral replication requires cellular components and processes because viruses extensively use host cell machinery for productive replication. Influenza viruses also influence cell-signaling pathways to evade the host’s immune system. Numerous host proteins are differentially expressed during IAV infection compared to non-infected cells. These differentially regulated proteins, which are probably required to support the viral life cycle or maintain host cell stress responses, are involved in different cellular pathways and functions such as host cell immunity, cell adhesion, signal transduction and transcription [5,6,7,8]. Several genome-wide RNAi screens have implicated host factors potentially involved in IAV replication [9,10,11].

IAV is a very well-studied virus. However, little is known about mechanisms of virus–host interactions during infection and disease progression. Different host proteins and different viral proteins play important roles in individual steps during the viral life cycle [12]. A better understanding of virus–host interactions will provide greater mechanistic elucidation of influenza virus replication, which may identify additional strategies to prevent or ameliorate infections. In the last two decades, many host proteins were found to interact with Influenza NP and RNA-dependent RNA polymerase complex (PA, PB1 and PB2) and some of these regulated the viral replication process [13,14,15,16,17,18].

Influenza A non-structural 1 (NS1) protein plays a major role in the production of high levels of viral proteins [19]. It is a multi-functional protein and its main role is to antagonize the host innate immune system (reviewed in [20,21,22]). Influenza viruses with truncated NS1 induced strong interferon (IFN) secretion and reduced morbidity in several animal models including swine, mice, and macaques [23,24,25]. NS1 protein inhibits the host’s RIG-I signaling cascade by blocking activation of transcriptional factors NFκB and IRF3, which are required for IFN transcription activation [26,27,28]. The NS1 effector domain (NS1-ED) binds PKR (protein kinase R) and inhibits its conformational changes, thereby inhibiting antiviral activity of IFN-induced PKR (reviewed in [26]). OAS (2’–5’ oligo A synthetase), an IFN-induced protein, is activated by dsRNA to produce poly-A chains that activate RNase L expression. RNase L can cleave viral ssRNA and thereby inhibit viral replication [29]. The NS1 RNA binding domain (NS1-RBD) binds dsRNA and inhibits the activation of the OAS/RNase L pathway [26,30].

Although NS1 proteins are not incorporated into virions, the high expression levels suggest that NS1 plays additional crucial roles during viral replication. According to the VirHostNet 2.0 [31] (May, 2016), 202 cellular proteins were detected and/or reported to interact with Influenza A NS1 [32]. Some interactome studies employed yeast 2-hybrid systems [33,34]. Many other interactome studies involved insertion of tag sequences such as TAP, Strep, Flag, V5, or FS into the NS1 sequence, and targeting the tags to co-immunoprecipitate NS1 and interacting host proteins, which were identified by mass spectrometry [35,36,37,38,39,40,41]. Inserting tag sequences may interfere with native protein structure and may mask epitopes. Therefore, we developed and characterized a panel of 9 different broadly cross reactive anti-NS1 monoclonal antibodies [42] and used them to identify host factors that interact with native IAV NS1 during natural IAV infection. We identified 124 novel putative NS1-interacting proteins and tested RNAi-mediated knockdown of most of these. Knockdown of the NS1-interacting nuclear mitotic apparatus protein 1 (NUMA1) had no effect on viral transcription or protein translation but significantly reduced infectious virus yield, suggesting NUMA1 plays important roles in IAV maturation.

## 2. Materials and Methods

### 2.1. Cells and Viruses

Human lung (A549; ATCC # CCL-185) and canine kidney (MDCK; ATCC # CCL-34) epithelial cells were cultured in complete Dulbecco’s modified Eagle’s medium (DMEM) supplemented with 10% and 5% fetal bovine serum (FBS), respectively. Human bronchial epithelial cells (HBEC-3KT; ATCC cat # CRL-4051; “HBEC”) were maintained at 37 °C in 5% CO_2_ in Airway Epithelial Cell Basal Medium (ATC PCS-300-030) supplemented with Bronchial Epithelial Growth kit (ATCC PCS-300-040). Influenza A virus (IAV) strains A/Puerto Rico/8/1934(H1N1) (PR8), A/WSN/1933(H1N1) (WSN), A/California/07/2009(H1N1) (pdm09), and A/New York/55/2004(H3N2) (NY55) were grown in MDCK cells by infecting at a multiplicity of infection (MOI) of 0.01 for 48 h. Some clones were concentrated at 64,000× *g* for 2 h at 4 °C.

### 2.2. Virus Titration

Serial 1:10 dilutions of viral stocks and experimental samples were titrated by plaque assay in MDCK cells, using a 1:1 mixture of 1.2% type 1 agarose and 2× DMEM, supplemented with 2.5 µg/mL Tosyl-L-lysyl-chloromethane hydrochloride (TLCK)-treated trypsin, as described [43]. Plates were incubated at 35 °C for 66 h, fixed with 2% formaldehyde and stained with crystal violet to determine viral plaque forming units (PFU) per mL.

### 2.3. Cytoplasmic and Nuclear Fractionation

A549 cells were infected with PR8 at a MOI of 5 PFU/cell. Mock-infected controls were treated similarly but without virus. Cells were harvested and processed as described [5] with minor modifications. Briefly, infected and mock-infected cells were scraped from plates at 6 and 24 h post infection (hpi), washed 3× with ice-cold phosphate buffered saline (PBS), cellular pellets resuspended in lysis buffer (150 mM NaCl, 10 mM Tris, pH 7.5, supplemented with 0.4% NP40 and 1× Roche complete™-ethylenediaminetetraacetic acid (EDTA)-free protease inhibitor, Mississauga, ON, Canada) on ice for 15 mins and vortexed every 5 min. Cytoplasmic extracts were prepared by centrifuging for 5 min at 500× *g*, nuclear pellets were re-extracted in lysis buffer supplemented with 8% sucrose, and supernatants combined for cytoplasmic fractions. The remaining pellets were washed 4× with PBS supplemented with 8% sucrose and 0.25× protease inhibitor. The 4×-washed pellets were resuspended in radioimmunoprecipitation (RIPA) buffer, sonicated 10 s, and nuclear extracts collected by centrifuging at 10,000× *g* for 10 min. The protein concentrations of all cytoplasmic and nuclear extracts were determined by a Pierce™ bicinchoninic acid (BCA) protein assay kit (Thermo Scientific, Waltham, MA, USA).

### 2.4. Co-Immunoprecipitation (Co-IP)

Cytoplasmic and nuclear lysates were initially pre-cleared with non-coupled protein G Dynabeads (Invitrogen, Waltham, MA, USA) for 90 min at 4 °C. The pre-cleared lysates were clarified at 10,000× *g* for 7 min. Dynabeads were washed 3× with TBST (Tris-buffered saline supplemented with 0.05% Tween 20) and a mixture of anti-NS1 mAbs 3F5, 5F4 and 4E10, which recognize different NS1 epitopes [42], was added to the beads. The mAbs and beads were incubated at room temperature for 90 min in a rotator to allow Ab-bead binding. Monoclonal α-Emprin (IgG2a), monoclonal α-SYN (IgG2b) and monoclonal α-HSA (IgG1) antibodies (gift from Dr. Wilkins, Manitoba Centre for Proteomics and Systems Biology) also were bound to Dynabeads to serve as isotype controls. Ab-coupled beads were washed 4× with TBST to remove unbound mAbs and mixed with the pre-cleared cellular fractions in a rotator overnight at 4 °C. The unbound fractions were discarded and beads were washed 4× and resuspended in TBST. The washed and resuspended bead-Ab-antigen complex represented immunoprecipitated (IP) products. Co-IPs were also performed after coupling anti-NUMA1 (Bethyl Laboratory, A301-510A), anti-PRPF19 (Bethyl Laboratory, A300-101A) and anti-UTP6 (Thermo Fisher, PA5-21716) antibodies to Dynabeads.

### 2.5. Processing of IP Product for Western Blot Analysis and Mass Spectrometry

The IP products and beads were washed 2× with RIPA buffer, 1× with ammonium bicarbonate buffer supplemented with 0.1% NP40 and resuspended in ammonium bicarbonate buffer. 10% of the resuspended bead mixtures were dissolved in sodium dodecyl sulfate (SDS) running buffer and resolved in 4–12% gradient Novex NuPAGE Sodium Dodecyl Sulfate Polyacrylamide Gel Electrophoresis (SDS-PAGE) Gels (Invitrogen, Waltham, MA, USA) for Western blot analysis and 90% of the resuspended beads were saved at −80 °C for subsequent mass spectrometry (MS) analysis. For MS analysis, the immunoprecipitated beads were digested overnight with 1 µg of trypsin in 100 mM ammonium bicarbonate solution at 37 °C. After tryptic digestion, equal volumes of trifluoroacetic acid (TFA)/Acetonitrile (ACN) (100% ACN & 1% TFA) were added to the digested IP products and vortexed 10–15 min. Digested peptides were separated from beads by centrifugation at 17,000× *g* for 5 min and were dried in a Savant SpeedVac vacuum dryer. Dried peptides were resuspended in 50 µL of 0.5% TFA and desalted with C18 ziptips. Eluted peptides were analyzed in an AB SCIEX (Concord, ON, Canada) Triple TOF 5600 mass spectrometer. Raw MS data were analyzed with Protein Pilot^TM^ 3.0 (ABSciex, Concord, ON, Canada). The proteins were identified based on cumulative peptide numbers and scores (cut-offs of a minimum of 2 peptides with unused score ≥2.0).

### 2.6. Transfection of Cells by siRNA

For initial screening, a reverse transfection format (RTF) SMART pool siRNA library was designed targeting 107 genes and purchased in 96-well format from Dharmacon (Lafayette, CO, USA). Reverse transfection of this siRNA array was carried out according to manufacturer’s protocol. In brief, sets of siRNA plates were rehydrated with transfection reagent/DharmaFECT-1 cell culture media and incubated for 60 min at room temperature. 4 × 10^3^ A549 cells were added to each well and incubated at 37 °C for 48 h to allow knockdown. Cell viability was determined in one set of plates with the Roche cell proliferation reagent WST1 and another set was infected with IAV PR8 at MOI 0.05 for 43 h. Cell viabilities of PR8-infected knockdown cells were also measured at the end of infection.

For specific gene knockdown, larger numbers of A549 cells were transfected with individual ON-Target plus siRNAs (Dharmacon) targeting NUMA1 gene or with scrambled non-targeting control siRNA (N-Si) according to the manufacturer’s protocol. Stock siRNAs and DharmaFECT^®^-1 transfection reagent (Dharmacon) were diluted separately with Opti-MEM^®^ I reduced serum medium (Life Technology, Waltham, MA, USA). A549 cells were transfected with siRNA every 24 h for 48 h. At 48 h post-transfection, cells were infected with IAV PR8 at MOI 0.05 or 3. Knockdown efficiencies of individual genes were checked by Western blot and IP using specific antibodies to relevant proteins.

### 2.7. Western Blot Analysis

Cell lysates obtained as described above were mixed with SDS electrophoresis sample buffer containing 90 mM DDT, boiled and subjected to electrophoresis in 4–12% gradient Novex NuPAGE SDS-PAGE Gels (Invitrogen). Resolved proteins were transferred to Immobilon-P polyvinylidene difluoride (PVDF) membranes (Millipore, Etobicoke, ON, Canada), and membranes were blocked in 5% skim milk and probed with various antibodies. Primary antibodies were monoclonal α-NS1 [42], monoclonal α-NP [44] (gift from Dr. Mingyi Li, National Microbiology Labs), monoclonal α-M1 (Thermo Fisher, MA1-80736), polyclonal α-M2 (Thermo Fisher, PA5-32233), monoclonal α-beta-actin (Cell Signalling, 3700S, Danvers, MA, USA), α-NUMA1 (Bethyl Laboratory, A301-510A, Montgomery, TX, USA), α-PRPF19 (Bethyl Laboratory, A300-101A) and/or α-UTP6 (Thermo Fisher, PA5-21716). Primary antibodies were detected with HRP-linked polyclonal α-mouse (Cell Signalling, 7076S), polyclonal α-rabbit (Cell Signalling, 7074S) or monoclonal VeriBlot™ (Abcam, Ab131366, Toronto, ON, Canada) secondary antibodies and HRP signals were detected using enhanced chemiluminescence (ECL) reagent (prepared in house). Images were taken with an Alpha Innotech Fluor Chem^®^ Q Imaging System and minimally processed by Adobe® Photoshop^®^ software (San Jose, CA, USA).

### 2.8. Immunofluorescent Microscopy

A549 cells were grown overnight in Nunc 8-well Lab-Tek chamber slides. The next day, cells were treated with individual siRNA. After 48 h knockdown, cells were infected at MOI 3, incubated 20 h, and fixed with 3.7% formaldehyde for 20 min. Fixed cells were washed 4× with PBS, permeabilized with 0.2% Triton-X 100 for 10 min, washed again 4× with PBS, blocked with 3% BSA/PBS for 1 h and probed with monoclonal mouse α-NS1 [42] and with polyclonal rabbit α-NUMA1 (Bethyl Laboratory, A301-510A). Slides were washed 4 more times with PBS, then treated with Alexa Fluor® 546 (dilution of 1:250, Invitrogen)-conjugated goat anti-mouse secondary antibody, with Alexa Fluor® 488 (dilution of 1:250, Invitrogen)-conjugated goat anti-rabbit secondary antibody in 1% BSA/PBS for 1 h at room temperature, and with 4’, 6-diamidino-2-phenylindole (DAPI) (Invitrogen, dilution of 1:10000). Slides were washed three times with PBS, a drop of mounting media was added (Pro Gold, Invitrogen) to each spot and images obtained with a Zeiss LSM710 laser-scanning microscope (Carl Zeiss MicroImaging GmbH, Oberkochen, Germany), using 20× and 40× objectives.

### 2.9. Structured Illumination Microscopy (SIM)

SIM was conducted according to the procedure described by Righolt et al. [45]. A549 cells were grown overnight on 18 × 18 mm high performance cover glasses with restricted thickness-related tolerance, D = 0.17 mm ± 0.005 mm (Zeiss, cat # 474030-9000-000). The next day, cells were treated with individual siRNA. After 48 h knockdown, cells were infected at MOI 3, incubated 20 h, and fixed with 3.7% formaldehyde for 20 min. Fixed cells were washed 4× with PBS, permeabilized with 0.2% Triton-X 100 for 10 mins, washed again 4× with PBS, blocked with 3% BSA/PBS for 1 hour and probed with rabbit polyclonal α-M2 or mouse monoclonal α-M1 antibodies diluted in 3% BSA/PBS for 6 h at 4 °C. Cover glasses were then washed 4× with PBT (PBS, 3% BSA and 0.05% Tween20), treated with Alexa Fluor 488-conjugated goat α-rabbit or Alexa Fluor 546-conjugated goat α-mouse secondary antibodies diluted in 1% BSA/PBS (1:250) for 1 h, washed 4× with PBT, treated with DAPI for 5 min and mounted with Vectashield or Prolong gold (Life Technology) mounting medium. The cover glasses were sealed with nail polish. Images were taken with a Zeiss ELYRA PS1 in SIM mode equipped with a Plan-Apochromat 63 ×/1.40 oil immersion objective and 2D and 3D images were processed as described [45].

### 2.10. Electron Microscopy (EM)

A549 cells were grown in P100 cell culture plates overnight and treated with individual siRNAs. At 48 h post-transfection, A549 cells were infected with PR8 at MOI 3. At 20 hpi, cells were harvested, and washed 3× with ice-cold PBS. Cell pellets were resuspended in EM grade Karnovsky fixative (2.5% glutaraldehyde and 4% paraformaldehyde in 0.1 M phosphate buffer, pH 7.2) and sent to the Histology Lab, Department of Human Anatomy and Cell Science, University of Manitoba for further processing. Images were obtained on a Philips CM10 Electron Microscope.

### 2.11. Real-Time Polymerase Chain Reaction (PCR)

PR8-infected A549 cells were harvested, washed 3× with cold PBS and total cellular mRNA was extracted with RNeasy Mini Kit (Qiagen, Venlo, Netherlands) according to the manufacturer’s protocol; 250 ng of purified mRNA was used to synthesize cDNA with the Go Script TM Reverse Transcription System kit (Promega, Madison, WI, USA). Real time polymerase chain reaction (PCR) was performed using Luminaries Color HiGreen High ROX qPCR kit (Thermo Fisher). According to the manufacture’s protocol, PCR master mix (10 µL) consisted of: 5 µL 2× Luminaries Color HiGreen High ROX qPCR master mix, 4.4 µL (100 ng) template cDNA and 0.3 µL each of 10 µM forward and reverse primers (Supplementary Table S2). PCR reactions were run in triplicate on an Applied Biosystems 7300 Real-Time PCR System. The program of cycle condition was 50 °C for 2 min, 95 °C for 2 min, and 50 cycles of (95 °C for 15 s and 60 °C for 30 s). The Ct values were normalized to 18S rRNA control and compared to non-targeting siRNA controls.

### 2.12. Bioinformatics and Statistical Analyses

Lists of IAV-NS1 interacting host proteins were generated from Protein Pilot analysis. The gene symbol and Uniprot IDs of all proteins were uploaded into the Database for Annotation, Visualization and Integrated Discovery (DAVID) [46] for functional tool analysis. NS1-interacting proteins were also uploaded into Consensus Path Database (CPDB) [47] for pathway and enrichment analysis. Groups of proteins identified in individual pathways in DAVID were uploaded into the Search Tool for the Retrieval of Interacting Genes/Proteins (STRING) [48] to visualize the protein-protein interactions. Interaction among viral proteins and host factors were also analyzed by using VirHostNet 2.0 [31] database. All statistical analyses were calculated in Microsoft-Excel and SigmaPlot^®^ software. *p*-values were determined using Student’s *t*-test.

## 3. Results

### 3.1. Co-Immunoprecipitation of Influenza A Virus (IAV) Non-Structural Protein 1 (NS1) from Infected Cells

**Figure 1:**
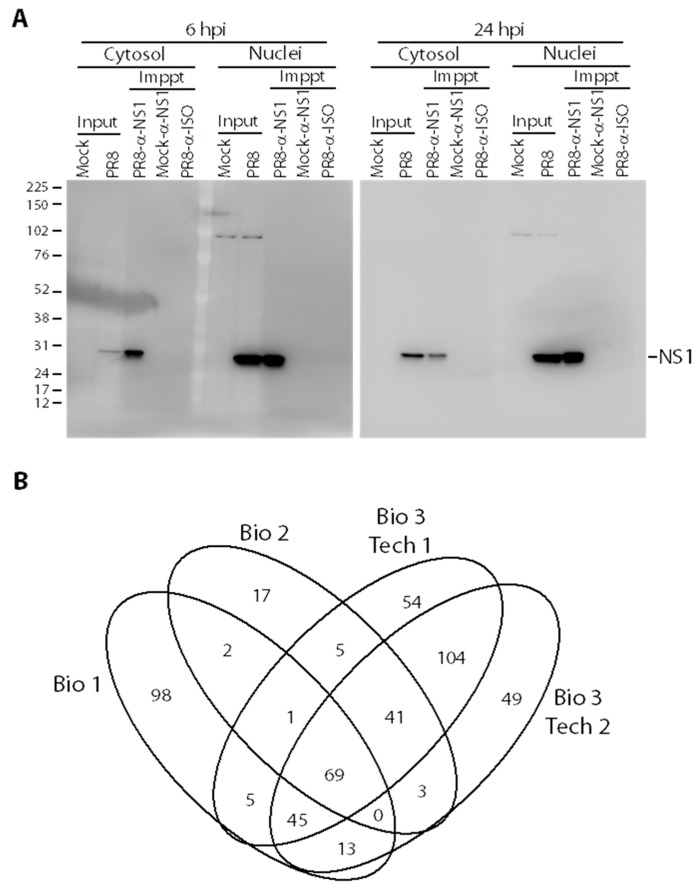
Identification of non-structural protein 1 (NS1)-interacting host proteins. (**A**) Western blot analyses of influenza A virus (IAV) NS1 immunoprecipitations. Samples were collected from the cytosols or nuclei of Mock- or PR8-infected cells in P150 dishes at indicated times and resolved in 10% sodium dodecyl sulfate polyacrylamide gel electrophoresis (SDS-PAGE) before (Input; 30 µg) and after (Immppt; 10% of total sample) treatment with Dynabeads to which the indicated antibodies (α-NS1 or Isotype-matched controls) had been pre-bound. Resolved proteins were transferred to Immobilon-P polyvinylidene difluoride (PVDF) membranes, probed with α-NS1 primary antibody, and re-probed with VeriBlot secondary α-mouse antibody. (**B**) Venn diagram indicating degree of overlap in protein identifications from 3 different biological replicates (Bio 1–3). Biological replicate #3 was repeated as 2 technical replicates (Bio 3 Tech1 and Bio 3 Tech 2).

We previously generated and characterized a panel of nine different broadly cross-reactive anti-NS1 monoclonal antibodies (mAbs) that detect five different epitopes on native and denatured forms of IAV NS1 [42]. For this NS1 interactome study, a mixture of anti-NS1 mAbs 3F5, 4E10 and 7D11, which recognize three of these different epitopes [42], were used, as described in Materials and Methods, to increase potential molecular interaction coverage. The anti-NS1 mAb mixture successfully pulled down NS1 protein (~26 kDa) from both PR8-infected cytosol and nuclei (Figure 1A). The 6 hpi nuclear input NS1 band intensity was higher than the cytosol input (Figure 1A), which correlated with previous observations of NS1 subcellular distribution [42]. No non-specific NS1 binding was seen in PR8-infected cytosolic or nuclear IPs with the isotype controls, nor in mock-infected control samples (Figure 1A).

### 3.2. Identifying NS1 Interacting Host Proteins by Mass Spectrometry (MS)

Once specific NS1 IP was confirmed, 90% of the IP products were processed and analyzed by MS for protein identifications. Three different biological replicates were performed and the last replicate was technically examined twice for a total of four replicates. Positive identification of NS1-interacting proteins was only considered when each protein was identified by at least 2 non-redundant peptides at unused scores ≥2 (*p* < 0.01). All protein IDs detected in the PR8-infected IP probed with isotype controls, and in mock-infected IP probed with anti-NS1 mAb mixture, were considered non-specific background binding and were subtracted from proteins in the PR8-infected IP probed with anti-NS1. 233, 138, and 324 NS1 interacting host factors were identified in the cytosolic and nuclear fractions of biological replicates 1, 2, and 3, respectively (Figure 1B). Overall, 183 unique NS1 interacting host factors were identified in at least two different biological replicates (Table 1 and Figure 1B). According to a recent analysis of the VirHostNet 2.0 [31] database, 59 of these 183 host factors (32%) had been previously reported to interact with IAV NS1 protein (indicated by # in Table 1), whereas 124 host proteins represent potentially novel NS1 interacting partners. PR8 viral matrix protein 1 (M1) and nucleoprotein (NP) were also detected in the NS1 IP with high peptide and high unused scores, confirming numerous other reports and their interaction with NS1 in the VirHostNet 2.0 database.

### 3.3. Bioinformatic Analysis of NS1 Interacting Proteins

**Figure 2:**
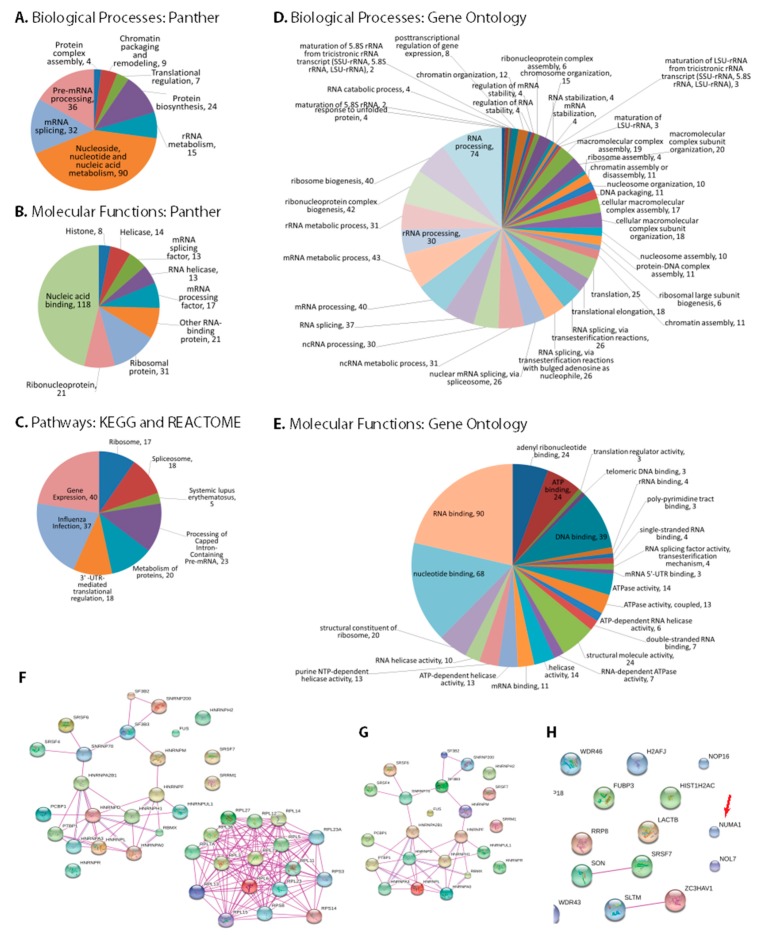
Pathway analyses of NS1 interacting host factors. DAVID-Panther analyses of (**A**) biological processes, (**B**) molecular functions and (**C**) reactome and Kyoto Encyclopedia of Genes and Genomes (KEGG) pathways. Gene Ontologies of (**D**) Biological processes and (**E**) Molecular functions. STRING analyses of NS1-interacting proteins in (**F**) Gene expression pathway, (**G**) Processing of capped intron-containing pre-mRNA pathway, and (**H**) Spliceosome pathway. Additional interacting protein networks are shown in Supplementary Figure S1A–D and a STRING interaction network of all 183 identified proteins is shown in Supplementary Figure S1E.

The multifunctional IAV-NS1 protein is expected to interact with a wide variety of host factors to serve its multiple functions. Therefore, categorizing the additional novel NS1-interacting host factors identified in our study may identify additional potential pathways and functions where NS1 plays vital roles during viral replication. DAVID and Panther analyses of the 183 genes showed various biological and molecular classes (Figure 2A,B). The top enriched biological processes were nucleoside, nucleotide and nucleic acid metabolism, mRNA splicing, pre-mRNA processing and protein biosynthesis. Similar molecular functions such as nucleic acid binding, mRNA splicing factor, mRNA processing factor and ribosomal protein were also enriched. The NS1-interacting host factors were involved in eight different pathways; gene expressions, 3′-UTR-mediated translational regulation, processing of capped intron-containing pre-mRNA, spliceosome, Influenza infection and metabolism of proteins (Figure 2C). Gene Ontology analyses of the 183 NS1 interacting host factors also showed diverse biological processes and molecular functions (Figure 2D,E). The top biological processes enriched were RNA processing, mRNA metabolic process, RNA splicing and translation. Some top molecular functions enriched were RNA binding, nucleotide binding, DNA binding and structural molecule activity.

Proteins in each identified GO pathway were visualized with STRING (Figure 2F–H and Supplementary Figure S1A–D) and all 183 proteins were collectively visualized to check their interaction network (Supplementary Figure S1E). Tight interactions among the proteins of different pathways, such as 3′-UTR-mediated translational regulation, ribosome and metabolism indicate the strong associations of these pathways with NS1 during Influenza virus replication.

### 3.4. Assessing the Necessity of NS1-Interacting Proteins for Viral Replication

**Figure 3:**
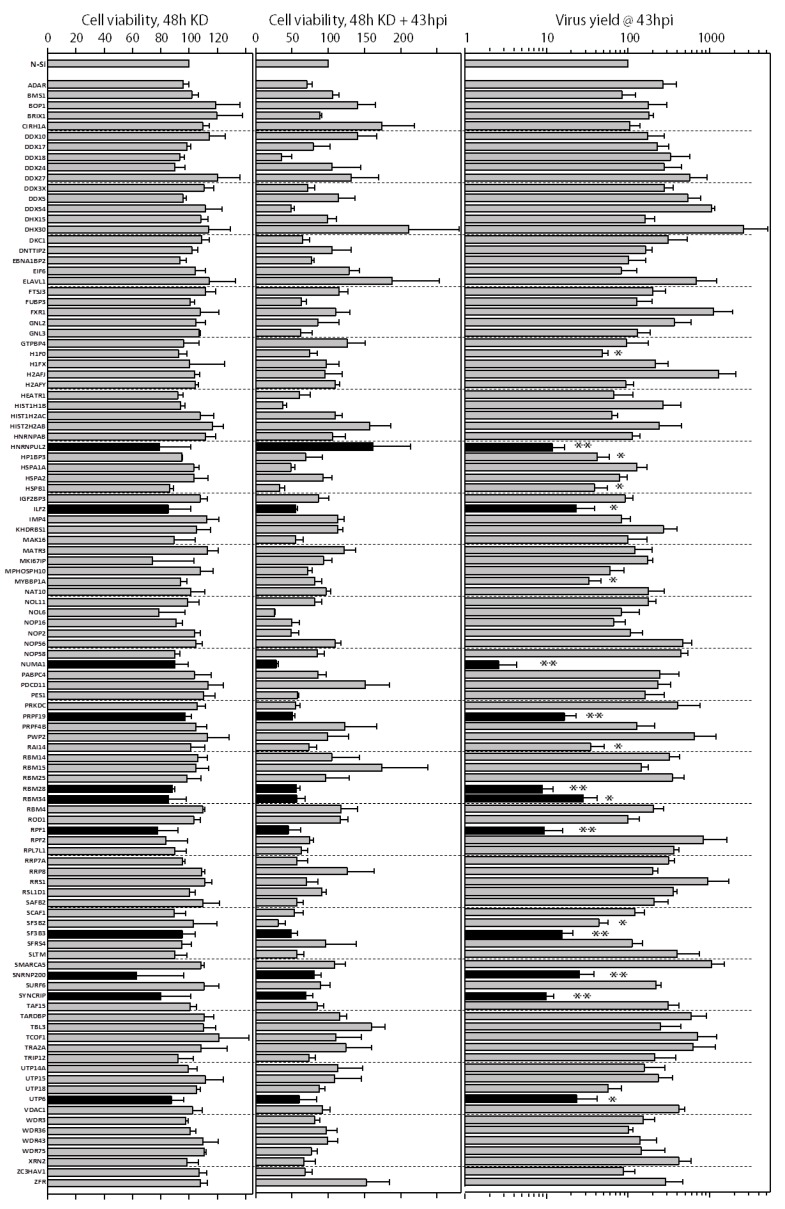
Genetic knockdown of candidate genes by siRNA array screen. Reverse transfections of indicated genes in A549 cells grown in 96-well plates were checked for cell viability with WST-1 at (**A**) 48 h after knockdown, and (**B**) after knockdown and PR8 infection at multiplicity of infection (MOI) = 0.05 for an additional 43 h. (**C**) Virus yields from PR8 infection after MOI = 0.05 infection at 43hpi were determined by plaque assay on canine kidney (MDCK) cells. All values were normalized to the corresponding non-silencing (N-Si) controls, which were set as 100%. Error bars represent standard error of the mean (SEM) from three independent replicates. *: *p* < 0.05; **: *p* < 0.005. The 11 genes, knockdown (KD) of which significantly reduced the infectious virus production to between 2.6–30% of the N-Si controls, are depicted as black bars, and cell viabilities after 91 h of knockdown, and the ratios of virus production to cell viability for these 11 genes are shown in Supplementary Figure S2.

In order to test the roles of these potentially novel NS1-interacting host factors, we designed a high-throughput, 96-well-based custom siRNA array; 107 proteins were targeted based on their novelty, functions and high MS scores. These 107 proteins were identified as NS1 interacting partners in 2 or more different biological replicates with at least 2 peptides and *p* < 0.05 (unused score ≥2.0). Cell viabilities were determined after 48 h of knockdown (KD) and most remained >80% viable compared to non-targeting control (N-Si) (Figure 3, left). The lowest viability (62.8%) was seen in *SNRNP200* siRNA treatment. The KD cells were infected with IAV PR8 at MOI 0.05 and the supernatants were harvested at 43 hpi to detect infectious viral yields to determine how efficiently the virus replicated. Cell viabilities after 48 h KD and 43 h infection were also measured (Figure 3, middle). Infected cell cultures remained >70% viable compared to N-Si. In 34 KD cases, PR8 infection reduced the cell viability to 69.6–26.9%. The lowest cell viability was seen in PR8-infected *NOL6* KD cells. In some KD cases, cell viabilities were highly increased after viral infection indicating that the KD may protect the cell from cytopathic effect (Figure 3, middle). Knocking down 11 genes significantly reduced the infectious virus production to between 2.6–30% of the N-Si controls (Figure 3, right). The lowest IAV yields were generated in *NUMA1* KD cells. Among these 11 KD gene candidates, *ILF2*, *HNRNPUL2* and *PRPF19* were previously detected as NS1-interacting factors in the VirHostNet 2.0 database. *NUMA1*, *RBM28*, *RBM34*, *RPF1*, *SYNCRIP*, *SF3B3*, *UTP6* and *SNRNP200* were novel discoveries, and the role of PRPF19 was recently reported [40] while our study was underway. These 11 gene candidates play roles in various biological processes, molecular functions and pathways (Supplementary Table S1). Some important biological processes and molecular functions include cellular macromolecular complex assembly and subunit organization; chromatin assembly, packaging and remodeling; nucleoside, nucleotide and nucleic acid metabolism; mRNA splicing; mRNA processing; nucleic acid binding; structural molecule activity; and ribonucleoprotein complex biogenesis. Some important pathways include spliceosome, gene expression and processing of capped intron-containing pre-mRNA.

### 3.5. Validation of NS1-Host Factor Interaction by Reciprocal Immunoprecipitation (IP)

**Figure 4:**
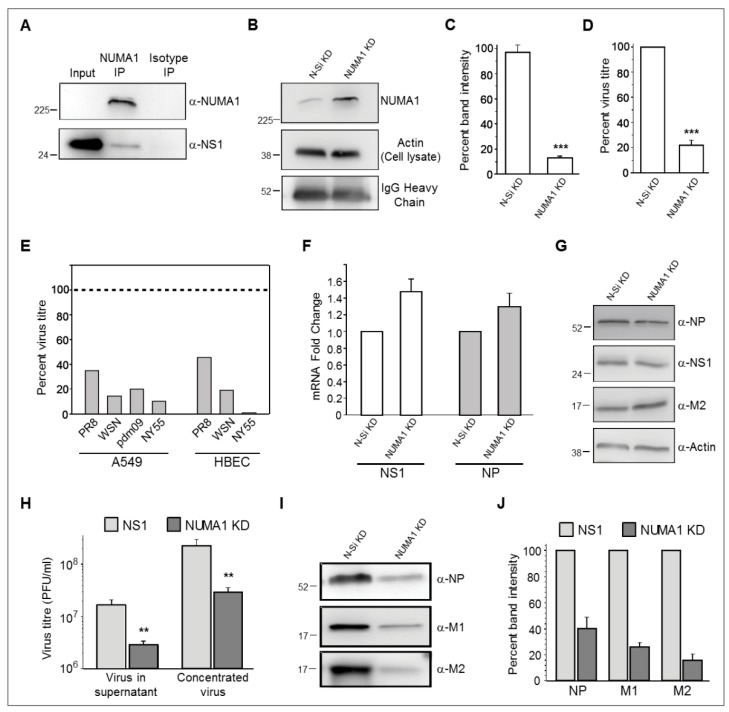
Characterizations of protein, RNA, and infectious virus production in NUMA1 KD cells. (**A**), Upper panel: confirmation that NUMA1 is immunoprecipitated and recognized by α-NUMA1 antibody in Western blot. NUMA1 could not be detected in non-concentrated cell lysates. Cell lysates were prepared from P100 dishes of A549 cells, reacted with Dynabeads to which α-NUMA1 Abs had been coupled, and ½ of the reaction dissolved in SDS-PAGE sample buffer, resolved in 10% SDS-PAGE, proteins transferred to Immobilon-P PVDF membranes, and probed with α-NUMA1 antibody. Lower panel: Cell extracts prepared from P100 dishes of A549 cells infected with PR8 at MOI = 5 PFU/cell were probed for NS1 prior to immunoprecipitation (Input; 30 µg), or were immunoprecipitated with beads to which NUMA1 or an irrelevant isotype control antibody had been bound. Co-precipitated products were resolved by SDS-PAGE and blots were immunoprobed with α-NS1 antibody. (**B**) Confirmation of NUMA1 KD efficiency in A549 cells. Sets of P100 dishes were treated with 25 nM of non-silencing (N-Si) control or with *NUMA1*-specific siRNA twice, 24 h apart for a total of 48 h treatment. Cell extracts were prepared and a 1/40th dilution probed for β-actin to confirm equivalent starting amounts (middle panel). Extracts were then immunoprecipitated with α-NUMA1-Dynabeads. After washing, beads were dissolved in SDS-PAGE sample buffer, proteins resolved by SDS-PAGE, and immunoprobed for NUMA1 (upper panel) or IgG heavy chain (lower panel). (**C**) Densitometry confirms NUMA1 was knocked down to ~16% of N-Si levels. (**D**) Percentages of infectious virus production from NUMA1 A549 KD cells compared to N-Si cells. Cells were infected at MOI of 0.05 and harvested at 43 hpi for plaque assay. (**E**) Percentages of indicated infectious IAV produced from *NUMA1* KD A549 and HBEC cells compared to N-Si cells at 43 hpi after MOI = 0.05 infection. The horizontal dashed line at 100% corresponds to each virus’ yield from matching N-Si cells. (**F**) mRNA levels of NS1 and of NP in A549 cells infected with PR8 at MOI = 5. *NUMA1* KD and N-Si cell lysates were quantified by real-time RT-PCR and normalized to both 18S RNA and to NUMA1 quantities produced in the N-Si cells. (**G**) Cell extracts prepared from N-Si- and *NUMA1* KD-infected cells were immunoprobed with the indicated viral proteins (right) or with actin. (**H**–**J**) Analyses of infected supernatants from N-Si or *NUMA1* KD cells, before ultracentrifugal concentration (**H**; left-most pair of bars) or after concentration (**H**; rightmost pair of bars, and **I** and **J**). Concentrated viruses were tested for infectivity (**H**) and immunoprobed for indicated structural proteins (**I**). (**J**) Densitometry confirms *NUMA1* KD cells produce particles with ~20–40% protein content compared to N-Si cells. Error bars represent SEM from two independent replicates. *: *p* < 0.05; **: *p* < 0.01; ***: *p* < 0.001.

Host factor NUMA1 was selected for reciprocal IP, based on its ability to significantly reduce viral replication (Figure 3, right), its functional properties such as biological process, molecular functions and pathways and the availability of antibodies. A549 cells were infected with IAV PR8 at MOI 5 and harvested at 24 hpi. Cellular proteins were extracted from harvested cells and analyzed by co-IP using Protein G Dynabeads coupled to anti-NUMA1 antibody. IP products were analyzed by Western blot analysis using both anti-NUMA1 and anti-NS1 Abs. No NUMA1 signal was detected in non-concentrated cell lysates but a strong NUMA1 signal was seen near the 225 kDa region in immunoprecipitated cell lysates (Figure 4A, upper panel). NS1 was also detected in the input cell lysate and in the NUMA1 IP but not in isotype control, which confirmed the NS1-NUMA1 interaction (Figure 4A, lower panel).

### 3.6. Role of Nuclear Mitotic Apparatus Protein 1 (NUMA1) in Influenza Virus Replication

Nuclear mitotic apparatus protein 1 (NUMA1) was detected in all four biological and technical NS1 interactome replicates (Table 1). In the initial siRNA screens, IAV production was significantly reduced compared to the N-Si in all independent studies (Figure 3, right), which indicated an important role for NUMA1 in IAV replication. To validate the siRNA array results, KD was performed in larger dishes of A549 cells with *NUMA1* Smart Pool siRNA (Dharmacon, Lafayette, CO, USA). Optimization experiments indicated sufficient KD (NUMA protein expression reduced to ~16%) when cells were treated twice with 25 nM siRNA 24 h apart and infected 48 h post-initial KD. Transfected A549 cells were ~100% viable at 48 h post-transfection compared to untreated A549. Comparative IP of *NUMA1*- and N-Si-KD cells, using identical concentrations of cell lysates, Protein G beads and anti-NUMA Ab, confirmed NUMA1 protein expression was reduced to ~15% of the N-Si treated cells (Figure 4B,C). A 1/40th dilution of input cell lysates confirmed cell lysate loading control (Figure 4B) and equivalent band intensities of anti-NUMA Ab heavy chains confirmed equal Ab treatment (Figure 4B). After optimization and KD validation, both *NUMA1* KD and N-Si cells were infected with IAV PR8 at MOI 0.05 and incubated 43 h. Knocking down *NUMA1* significantly reduced infectious PR8 production to ~21% compared to the N-Si in four independent experiments (Figure 4D), which validated the *NUMA1* KD results from the siRNA array.

### 3.7. NUMA1 Knockdown (KD) Does not Affect IAV Transcription and Translation

Since infectious virus production was consistently reduced in *NUMA1* KD cells, we next tested specific steps in virus replication. NUMA1 is mainly a nuclear protein, but it is also found in the cytoplasm [49]. After infection, viral ribonucleoproteins (vRNPs) need to enter the nucleus for transcription. We initially checked whether the import of incoming vRNPs or the transcription process was inhibited in *NUMA* 1 KD cells by analyzing viral RNA production. *NUMA1* KD and N-Si KD cells were infected with IAV-PR8 at MOI 3, infected cells were harvested at 18 hpi and total RNA was extracted. Synthesis of viral NP and NS1 RNAs were not reduced in *NUMA1* KD cells (Figure 4E). Thus, *NUMA1* KD did not affect early vRNP entry into the nucleus and transcription of NS1 and NP. After transcription, viral mRNAs are transported to the cytoplasm and translated by host cell machinery. Whole cell lysates were extracted from PR8-infected *NUMA1* KD and N-Si cells at 18 hpi and analyzed by Western blot. Early (NS1 and NP) and late (M2) viral protein translation was not reduced in the infected *NUMA1* KD A549 cells compared to the N-Si cells (Figure 4F). Therefore, *NUMA1* KD did not impede the transport of viral mRNAs to the cytoplasm and the translation process.

### 3.8. NUMA1 KD Affects IAV Maturation

Although viral proteins were synthesized efficiently in *NUMA1* KD A549 cells, infectious virus production was significantly reduced (Figure 4D). To determine the generality of this observation, we then knocked down *NUMA1* in human bronchial epithelial cells (HBEC-3KT; ATCC cat # CRL-4051; “HBEC”), and in additional A549 cells, and determined the capacity of additional H1N1 and H3N2 IAV strains to replicate in these KD cells. All tested IAV clones were reduced 55–99% in the *NUMA1* KD cells compared to non-silencing control cells (Figure 4E). These data suggest that NUMA1 depletion might affect infectious virus production. To examine this possibility, *NUMA1* KD and N-Si cells were infected with PR8 at MOI 0.05 and supernatants were harvested at 43 hpi. Viruses were pelleted from the N-Si and *NUMA1* KD supernatants by ultracentrifugation and resuspended in small equal volumes of PBS. Titration of the pre- and post-concentrated viruses confirmed that viral titers from the *NUMA1* KD cells were significantly reduced to ~20% of titers from N-Si cells (Figure 4H). To differentiate between the possibilities that equal numbers of particles were released from the *NUMA1* KD cells but they were less infectious than particles released from N-Si-treated cells, or that fewer particles were matured from *NUMA1* KD cells, equal volumes of purified and resuspended viruses generated from the N-Si and *NUMA1* KD cells were analyzed by Western blot to detect viral structural proteins NP, M1 and M2. The amounts of all tested proteins from *NUMA1* KD cells were reduced to 40%, 26% and 16%, respectively compared to viral proteins produced from the N-Si (Figure 4I,J). Therefore, the average structural protein reduction was similar to the infectivity reduction (Figure 4D,E,I,J), suggesting no significant difference in particle-to-PFU ratios, but that NUMA1 is involved in viral maturation.

**Figure 5:**
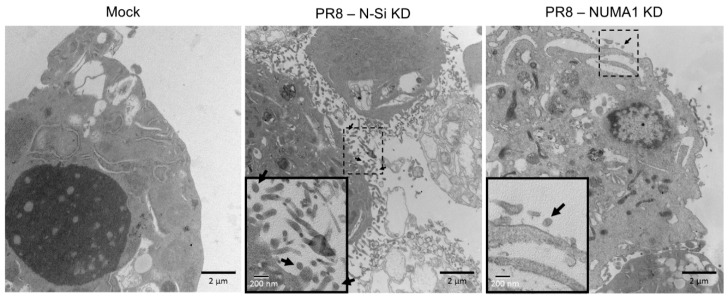
Ultrastructural examination of Mock-infected, and of PR8-infected N-Si and *NUMA1* KD cells at 20 hpi after MOI = 3 infection. Mock-infected, and N-Si and *NUMA1* KD PR8-infected, A549 cells were harvested, processed with EM Grade Karnovsky fixative and stained with uranyl acetate. All processed samples were analyzed with a Philips CM-10 electron microscope by the histology lab, Department of Human Anatomy. Numerous ~100 nm particles appear to be budding from infected N-Si KD cells (black arrows, center panel) whereas virus production was significantly reduced in PR8-infected *NUMA* KD A549 cells (right panel). Boxed regions are enlarged in the lower left insets and scale bars for the micrographs, and for the insets, are indicated.

As an alternate method to assess differences in virus maturation, we infected non-silencing and *NUMA1* KD cells with PR8 at MOI = 3 and examined infected cells at 20 hpi by electron microscopy. Numerous small spherical structures, consistent with the ~100 nm IAV virion size, were clearly visible outside but near the cell membrane, presumably after budding (Figure 5, middle, arrows) in N-Si infected cells. In contrast, production of these virus-sized particles was greatly reduced in infected *NUMA1* KD cells (Figure 5, right), in agreement with previous observations of reduced extracellular viral protein and infectious virus from these *NUMA1* KD cells.

### 3.9. Localization of NUMA1 and Viral NS1, M1 and M2 Proteins in NUMA1-Deficient Cells

**Figure 6:**
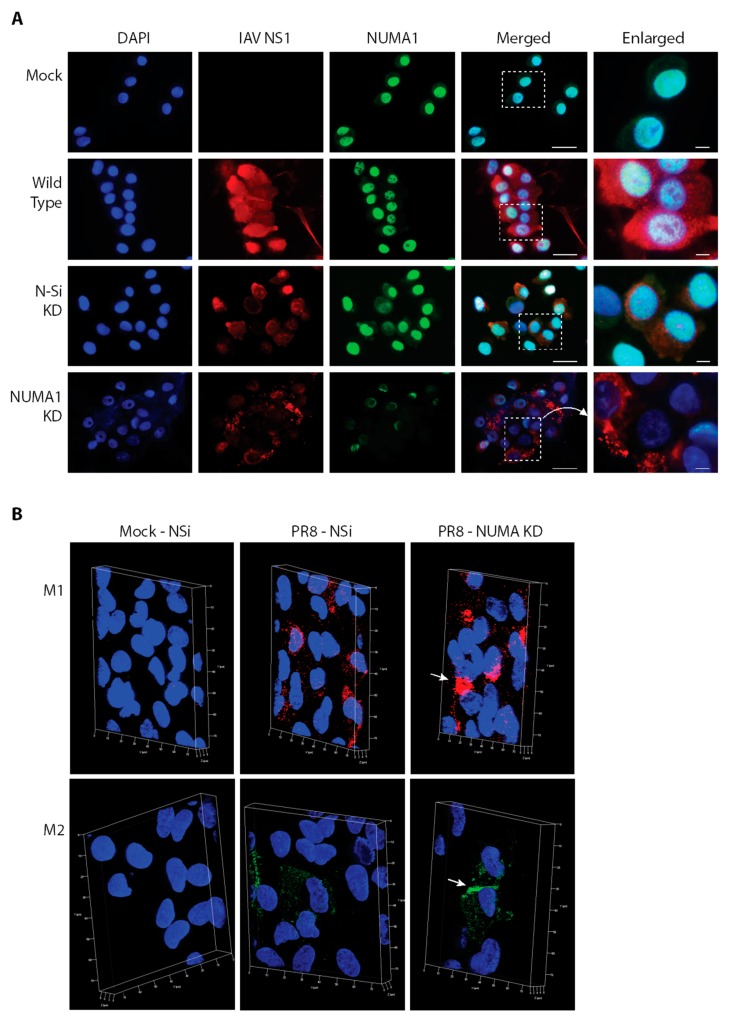
Immunofluorescent localization of cellular NUMA1 and of viral NS1, M1 and M2 proteins in A549 cells infected with PR8 at MOI = 3. (**A**), Mock (top row), non-KD infected wild-type (2nd row), non-silencing KD infected (N-Si; 3rd row), and infected *NUMA1* KD cells (bottom row) were stained with 4’, 6-diamidino-2-phenylindole (DAPI) to detect nuclei (left-most column; blue), with anti-IAV NS1 (2nd column; red), and with anti-NUMA1 (3rd column; green). Merged images are shown in the 4th column and boxed regions are enlarged at far right. The NUMA1 merged box was rotated clockwise 90°. Scale bars are 25 µm for the low-magnification images and 5 µm for the enlarged images. (**B**), Mock infected N-Si (left), PR8-infected N-Si (middle) and PR8-infected *NUMA1* KD (right) A549 cells were stained with Alexa Fluor 546 anti-M1 (red; top) or with Alexa Fluor 488 anti-M2 (green; bottom) and analyzed by super resolution structured illumination microscopy (SIM) at 20 hpi. Nuclei were stained with DAPI (blue). White arrows indicate the cluster of M1 in *NUMA1* KD cells (top, right) or accumulation of M2 near cytoplasmic membranes of *NUMA1* KD cells (lower, right). Scale bars represent 10 µm for the *X* and *Y* axes, and 2µm for the *Z* axis, respectively.

To further assess *NUMA1* KD and to determine localization of various viral non-structural and structural proteins under our control and KD conditions, we infected A549 cells with PR8 at MOI = 3 and examined cells for various protein markers. Viral NS1 protein was found in both nuclei and dispersed throughout the cytoplasm in most infected cells, but was primarily localized to perinuclear regions in the *NUMA1* KD cells (Figure 6A). NUMA1 was found predominantly in nuclei, co-localizing with DAPI, except in the *NUMA1* KD cells where the signal intensity was clearly reduced and the protein occupied only some of a few nuclei. As expected from the initial identification of NUMA1 (Table 1) and reciprocal immunoprecipitations (Figure 4) indicating NS1/NUMA1 interaction, NS1 and NUMA1 co-localized in the nuclei as indicated by the pale blue/white color in wild-type and N-Si KD cells. However, this interaction was greatly reduced in the *NUMA1* KD cells as indicated by the few pale blue/white patches (Figure 6A, bottom right).

The IAV M1 protein plays important roles in assembly of progeny virions. It helps in transporting vRNPs from the nucleus to the budding sites [50,51]. Therefore, we determined whether virus production was reduced due to the relocation of viral M1 in infected *NUMA1* KD cells. To investigate this, *NUMA1* KD and N-Si KD A549 cells were infected with PR8 at MOI 3, fixed at 20 hpi, treated with anti-M1 Ab and analyzed by super resolution structured illumination microscopy (SIM). M1 proteins accumulated and formed clusters adjacent to the nucleus in infected-*NUMA1* KD A549 cells (Figure 6B (upper right), white arrow). However, similar M1 clustering was not observed in PR8-infected N-Si cells, where M1 was more evenly distributed in extra-nuclear regions (Figure 6B (upper middle)). Thus, the SIM results suggested that M1 protein trafficking was interrupted in *NUMA1* KD cells and NUMA1 may be involved in M1 trafficking. M2 is another IAV protein that plays important roles in viral assembly and budding steps near the cytoplasmic membrane [50,52]. SIM was also conducted with PR8-infected *NUMA1* KD and N-Si cells. In NUMA1 depleted cells, most of the M2 proteins accumulated near the plasma membrane (Figure 6B (lower right), white arrow). However, M2 proteins were more evenly distributed in extra-nuclear regions in PR8-infected N-Si cells (Figure 6B (lower middle)).

## 4. Discussion

The goal of this study was to detect novel host factors that interact with native IAV NS1 during natural infection and viral replication. To target a wide range of NS1-interacting host factors during viral replication, we selected early and late time points. We had previously detected NS1 as early as 5–6 hpi [42]. Therefore, we selected 6 and 24 hpi as early and late time points for our co-IP experiments. 183 NS1-interacting host proteins were detected in at least two different biological replicates and most of these are involved in different cellular biological processes, molecular functions and pathways.

IAV utilizes the host cell system for its gene expression. To initiate IAV transcription, viral RNA-dependent RNA polymerase (RdRp) executes cap-snatching from host pre-mRNA and NS1 may play roles during this step [20,53]. Influenza viruses use the host’s splicing machinery to produce M1, M2, NS1 and NS2/NEP proteins [54,55]. NS1 interacts with spliceosomal subunits U2 and U6 during viral replication, potentially favouring their splicing [56,57]. The 5’ UTR of viral mRNAs can mediate selective viral mRNA translation over cellular mRNA translation [20,58,59]. NS1 plays important roles in initiating viral mRNA translation without affecting host mRNAs [60]. NS1 interacts with the 5’ UTR of viral mRNAs and with translation initiation factors PABP1 and eIF4GI, which enhance viral mRNA translation [20,58,61,62]. NS1 also interacts with factors involved in mRNA export, such as NXF1/Tap, and blocks host mRNA export [63]. In addition, NS1 interacts with viral RNP and regulates IAV replication [62,64]. Numerous NS1-interacting host factors we identified were enriched in gene expression, splicing, protein metabolism, nucleic acid binding, translational regulation and mRNA processing (Figure 2A–E). IAV may use these host factors to favour viral replication. We also identified large numbers of RNA-binding proteins. NS1 contains two functional domains: an N-terminal RNA-binding domain and C-terminal effector domain. The RNA binding and effector domains interact with different cellular RNAs and host factors, respectively [20,26,65], potentially accounting for the large numbers of RNA binding proteins we identified. 

Many of our NS1-interacting host proteins interact with each other according to STRING analysis (Figure 2F–H). Thus, some of the newly identified proteins may not directly interact with NS1 but may have been pulled down in the IPs by secondary interactions. However, we validated the interactions of NUMA1 with IAV-NS1 by reciprocal co-IP (Figure 4A), identifying NUMA1 as one of several proteins not currently known to interact with other identified proteins (Supplementary Figure S1, red circle) according to STRING, suggesting that the NUMA1-NS1 interaction is a direct one.

We knocked down 107 of the most promising NS1-interacting host proteins to evaluate their impact on IAV replication. Several groups have used viral gene luciferase assays or viral protein immunostaining in 96 well formats to detect viral replication, but these are surrogates for RNA production or protein, respectively, and do not address progeny viral infectivity directly. We measured viral titers to assess virus replication and infectious virus production directly from infected-KD cells. Knocking down 11 NS1-interacting host proteins significantly reduced IAV replication at least 3-fold compared to the control cells in three different experimental replicates. Although some genes’ KD, such as *DHX30*, *DDX54*, *ELAVL1*, *FXR1*, *H2AFJ*, *NOP56* and *RRS1* increased viral titers (Figure 3C), we focused this study on the 11 gene candidates that significantly reduced virus titer. Most cells were ≥80% viable after 48 h KD; however, many infected-KD cells were less viable 43 h later (Figure 3B). Influenza causes virus-induced cell death during viral replication [66,67,68]. To differentiate between virus-mediated versus prolonged KD-mediated cell death, we examined cell viabilities of non-infected cells at 91 h post KD (Supplementary Figure S2A). Viability of these cells was uniformly higher than for the KD-infected cells, indicating loss of cell viability at prolonged time was primarily caused by viral-induced cell death. Lower viral titer in many of the KD cells also corresponded with lower cell viability post-infection. For example, *ILF2* KD resulted in ~50% cell viability at the end of infection. Therefore, we compared viral yields to post-infection cell viabilities for the N-Si and all 11 KD A549 cells. Even when accounting for reduced cell viability, viral yields were substantially lower in many of the KD cells (Supplementary Figure S2B), including *NUMA1* KD, suggesting the lower titers were caused by the KD, not by the lower cell viability. Among these 11 candidates, ILF2, HNRNPUL2, and PRPF19 were previously identified as NS1 interacting proteins in the VirHostNet 2.0 database. The PRPF19 deficient cells were demonstrated to reduce influenza virus production in a recent study [40] while our study was ongoing. NUMA1, RAI14, RBM28, RBM34, RPF1, SF3B3, SNRNP200, and UTP6 proteins represent newly discovered potential NS1 interacting proteins, knocking down of which significantly reduces infectious IAV production. These novel NS1-interacting host factors may have roles in controlling viral/host mRNA maturation, splicing and gene expression to favour viral replication.

NUMA1, also known as NuMA, is an important structural component of both the nucleus and spindle poles, and it plays essential roles during the assembly and maintenance of the mitotic spindle in the cell cycle (reviewed in [69]). The NuMA protein is solubilized and modified extensively, including phosphorylation at unknown sites, during Herpes Simplex Virus (HSV) infection [70]. Knocking down NuMA expression in Hep-2 cells also decreased HSV production. NUMA1 has strong connections with microtubules. During mitosis, NUMA1 plays an important role in connecting the microtubules to the spindle poles [69,71] Tubulin (a monomer of microtubules) directly binds with the C-terminus of NUMA1 [72]. Other studies suggested that NUMA1 binds with microtubules in association with dynein, a microtubule motor protein [73,74]. Microtubules and dynein contribute in transporting newly synthesized proteins through the Golgi complex in the exocytic pathway [75,76] to the cell surface.

*NUMA1* KD significantly reduced replication of several IAV strains in both A549 and HBEC cells (Figure 4E), which has not been previously reported. We found no significant inhibition of PR8 viral mRNA synthesis in NUMA1 deficient cells compared to the control cells (Figure 4F,G). This suggests that viral entry into the cell, import of incoming vRNPs to the nucleus and viral transcription steps do not depend on NUMA1 proteins. NUMA1 deficient cells and N-Si control cells synthesized similar amounts of IAV structural and non-structural proteins, which indicates that viral mRNA transport to the cytoplasm and translation were not affected by *NUMA1* KD (Figure 4H). The levels of IAV structural proteins released into the supernatant were significantly reduced in *NUMA1* KD cells compared to the N-Si control cells (Figure 4I,J). Thus, viral transcription and translation steps are not inhibited, but viral maturation steps are inhibited within NUMA1 deficient cells.

During viral maturation, newly produced vRNPs need to be exported to the cytoplasm, and then transported to the cell membrane for progeny virus assembly and budding. Influenza M1 directly interacts with vRNPs and plays vital role in exporting these vRNPs into the cytoplasm [51,77]. The viral envelope proteins, HA, NA and M2, are independently transported to the cell membrane, where eventual interactions among the viral proteins and vRNPs lead to progeny virion assembly and budding. M1 acts as a linker between vRNPs and envelope proteins (reviewed in [51,78]). In addition, our previous study showed NS1 proteins were detected in the nucleus at early times of infection and later spread into the cytoplasm [42]. HA, NA and M2 use exocytic pathways to reach the cell membrane for assembly and budding through the *trans* Golgi network [51,79]. After the export of vRNPs-M1 complexes from the nucleus to the cytoplasm, it is suggested that the vRNPs-M1 complexes can be transported to the cell membrane by piggy-backing on the HA and NA cytoplasmic domains or via cytoskeleton elements [51,52,80,81,82]. Our SIM images of PR8-infected *NUMA1* KD cells showed IAV M1 proteins formed clusters within the cytoplasmic regions, in close proximity to the nucleus, whereas no clustering was seen within the N-Si cells (Figure 6B). In PR8-infected *NUMA1* KD cells, NP levels were also higher than in the PR8-infected N-Si cells (Figure 4H). Therefore, transport of M1-associated viral proteins to the cell membrane assembly and budding site was inhibited in NUMA1 deficient cells, which ultimately reduced the infectious virus production compared to the N-Si cells. However, an increase of IAV M2 proteins was also deposited near the *NUMA1* KD cytoplasmic region (Figure 6B), which suggested that M2 was produced but could not participate in viral maturation with other structural proteins. EM results also showed significant reduction of virus budding in *NUMA1* KD cells compared to the N-Si cells (Figure 5).

**Figure 7:**
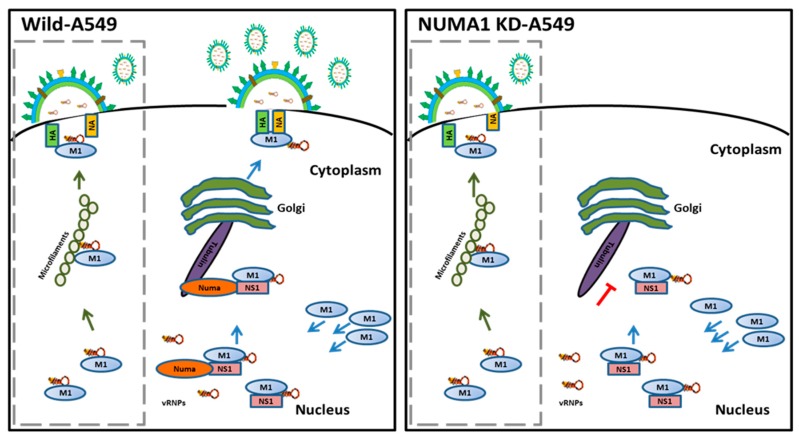
Proposed model for the role of NUMA1 during IAV replication. Major trafficking pathway, involving NUMA1, tubulin and exocytosis via the Golgi are indicated in the right of each panel. Lack of NUMA1 on the right results in significant attenuation of this pathway. An alternate, presumably minor pathway involving microfilaments (left side of each panel in dashed box), accounts for minor amounts of progeny virus production in normal, wild-type infection and in *NUMA1* KD cells.

We propose a model illustrating the role of NUMA1 in IAV maturation (Figure 7), in which the viral M1-RNP complexes transport from the nucleus into the cytoplasm and M1 interacts with NS1. 

We report in the current study that NS1 interacts with host NUMA1 protein during IAV replication. NS1 works as a bridge between NUMA1 and M1 proteins. After that, the strong link between NUMA1 and microtubules facilitates the transfer of viral M1-associated proteins (vRNPs) to the cytoplasm using the exocytic pathway via trans-Golgi network for assembly and budding. We also propose that M1-vRNPs are not able to interact with the microtubule network in NUMA1 deficient cells; thus, the transportation of these essential proteins to the assembly site is obstructed. IAV RNPs can be routed to the cell periphery through the Rab11-dependent vesicular transport system [83]. Moreover, M1 and NP vRNP also can reach the assembly site through cytoskeletal microfilaments [82]. These Rab11 and microfilament-mediated transport systems might contribute to the generation of some viruses (2.6–20% of the N-Si) in our PR8-infected *NUMA1* KD cells. Our proposed model elucidates the mechanism performed by NUMA1 protein to help influenza virus replication by the exocytic pathway.

In conclusion, novel and essential NS1-interacting host factors including NUMA1 identified in this study shed further light on the detailed mechanism of influenza virus replication and may identify alternative non-viral targets to develop new antiviral therapies.

## Figures and Tables

**Table 1:** NS1-interacting host proteins identified in at least 2 biological experiments.

Uniprot	Gene Symbol	Protein	Peptides (95%)				Unused Score *			
			Bio1	Bio2	Bio3 Tech1	Bio3 Tech2	Bio1	Bio2	Bio3 Tech1	Bio3 Tech2
P55265	**ADAR**	Double-stranded RNA-specific adenosine deaminase	4	4	20	20	2.86	7.54	37.77	35.31
Q14692	**BMS1**	Ribosome biogenesis protein BMS1 homolog	3	9	7	5	4.29	17.6	16.75	10.34
Q14137	**BOP1**	Ribosome biogenesis protein BOP1	4	9	7	9	8	17.8	14.19	15.1
Q8TDN6	**BRIX1**	Ribosome biogenesis protein BRX1 homolog	2	5	8	10	3.54	11.06	15.72	16.79
Q9Y224	C14orf166#	UPF0568 protein C14orf166	3	-	8	9	6	-	13.56	14.89
Q1ED39	C16orf88	Protein C16orf88	-	4	6	8	-	8	13.53	16.01
Q9Y3I0	C22orf28	UPF0027 protein C22orf28	6	-	12	10	9.47	-	20.52	21.07
Q7Z7K6	CENPV	Centromere protein V	4	2	3	3	7.76	2.96	5.84	5.67
Q969X6	**CIRH1A**	Cirhin OS	-	10	6	11	-	18.31	13.36	19.31
Q13206	**DDX10**	Probable ATP-dependent RNA helicase DDX10	-	2	4	3	-	2.29	8.05	5.44
Q92841	**DDX17#**	Probable ATP-dependent RNA helicase DDX17	5	2	18	16	7.4	4.03	29.5	28.76
Q9NVP1	**DDX18**	ATP-dependent RNA helicase DDX18	9	14	12	13	17.53	20.77	22.19	20.23
Q9NR30	DDX21#	Nucleolar RNA helicase 2	14	5	14	16	27.57	10.05	25.03	30.43
Q9GZR7	**DDX24**	ATP-dependent RNA helicase DDX24	4	4	11	8	8.15	7.07	20.93	15.19
Q96GQ7	**DDX27**	Probable ATP-dependent RNA helicase DDX27	-	4	5	5	-	9.11	10.71	10.45
O00571	**DDX3X#**	ATP-dependent RNA helicase DDX3X	4	-	9	12	8	-	14.93	18.22
P17844	**DDX5**	Probable ATP-dependent RNA helicase DDX5	3	-	-	7	5.88	-	-	11.15
Q8TDD1	**DDX54**	ATP-dependent RNA helicase DDX54	2	6	2	2	4	11.81	3.78	3
Q9NY93	DDX56#	Probable ATP-dependent RNA helicase DDX56	-	4	-	2	-	7.22	-	4.45
O43143	**DHX15#**	Putative pre-mRNA-splicing factor ATP-dependent RNA helicase DHX15	5	-	28	22	8.66	-	46.27	40.65
Q7L2E3	**DHX30#**	Putative ATP-dependent RNA helicase DHX30	2	9	53	59	3.05	17.57	87.85	100.67
O60832	**DKC1**	H/ACA ribonucleoprotein complex subunit 4	3	3	5	3	4.52	6.14	10.8	6.18
Q5QJE6	**DNTTIP2**	Deoxynucleotidyltransferase terminal-interacting protein 2	-	8	5	4	-	14.42	10.49	8.82
Q99848	**EBNA1BP2**	Probable rRNA-processing protein EBP2	2	7	7	7	3.5	11.2	12.59	13.09
P19525	EIF2AK2	Interferon-induced, double-stranded RNA-activated protein kinase	-	3	6	6	-	6.6	12.89	12.21
P56537	**EIF6**	Eukaryotic translation initiation factor 6	2	6	4	5	2.6	9.54	4.01	5.14
Q15717	**ELAVL1#**	ELAV-like protein 1	5	9	15	16	7.14	19.31	24.84	23.5
Q8IY81	**FTSJ3**	Putative rRNA methyltransferase 3	5	16	10	8	9.87	30.25	21.22	15.88
Q96I24	**FUBP3**	Far upstream element-binding protein 3	3	3	11	11	4.21	6.85	21.46	20.94
P35637	FUS#	RNA-binding protein FUS	4	-	3	3	7.38	-	4	5.4
P51114	**FXR1#**	Fragile X mental retardation syndrome-related protein 1	2	-	3	2	4.01	-	2.9	2.68
Q13823	**GNL2**	Nucleolar GTP-binding protein 2	-	3	2	2	-	4.88	2.57	3.41
Q9BVP2	**GNL3**	Guanine nucleotide-binding protein-like 3	-	4	4	4	-	9.03	8.98	8.39
Q9BZE4	**GTPBP4**	Nucleolar GTP-binding protein 1	5	9	14	14	9.64	18.5	26.81	25.87
P07305	**H1F0#**	Histone H1.0	-	3	5	3	-	4.23	6.25	4.09
Q92522	**H1FX**	Histone H1x	-	2	2	2	-	4.36	3.34	2.82
Q9BTM1	**H2AFJ**	Histone H2A.J	-	2	2	-	-	2.8	4.4	-
Q71UI9	H2AFV	Histone H2A.V	-	2	5	4	-	3.35	6.96	6.22
O75367	**H2AFY**	Core histone macro-H2A.1	2	3	5	8	5.1	4.84	9.07	14
Q9H583	**HEATR1**	HEAT repeat-containing protein 1	7	14	14	9	12.41	27.31	26.58	18.09
P16401	**HIST1H1B#**	Histone H1.5	4	6	10	8	6.05	9.63	17.07	14
Q93077	**HIST1H2AC**	Histone H2A type 1-C	-	4	2	-	-	6.87	4.27	-
Q8IUE6	**HIST2H2AB**	Histone H2A type 2-B	-	4	11	11	-	8.13	10.21	12.76
Q13151	HNRNPA0	Heterogeneous nuclear ribonucleoprotein A	4	3	5	5	5.07	4.36	9.15	8.94
P22626	HNRNPA2B1	Heterogeneous nuclear ribonucleoproteins A2/B1	3	3	11	15	6.66	5.21	18.47	23.97
P51991	HNRNPA3#	Heterogeneous nuclear ribonucleoprotein A3	4	-	8	37	8.06	-	17.75	58.02
Q99729	HNRNPAB#	Heterogeneous nuclear ribonucleoprotein A/B	3	-	3	3	3.2	-	3.05	2.57
Q14103	HNRNPD	Heterogeneous nuclear ribonucleoprotein D0	-	2	3	4	-	4	5.38	7.08
P52597	HNRNPF#	Heterogeneous nuclear ribonucleoprotein F	3	-	16	14	5.21	-	18.27	19.02
P31943	HNRNPH1	Heterogeneous nuclear ribonucleoprotein H	6	4	-	-	7.82	6.59	-	-
P55795	HNRNPH2#	Heterogeneous nuclear ribonucleoprotein H2	2	-	10	-	4	-	12.4	-
P31942	HNRNPH3	Heterogeneous nuclear ribonucleoprotein H3	2	-	3	2	2.6	-	5.07	4.08
P14866	HNRNPL#	Heterogeneous nuclear ribonucleoprotein L	-	30	33	36	-	32.72	28.44	32.15
P52272	HNRNPM#	Heterogeneous nuclear ribonucleoprotein M	12	6	26	26	16.03	11.55	37.44	32.3
O43390	HNRNPR#	Heterogeneous nuclear ribonucleoprotein R	13	12	27	27	19.81	21.8	43.09	37.93
Q9BUJ2	HNRNPUL1#	Heterogeneous nuclear ribonucleoprotein U-like protein 1	7	5	10	9	13.72	10.45	13.58	17.97
Q1KMD3	**HNRNPUL2#**	Heterogeneous nuclear ribonucleoprotein U-like protein 2	5	7	19	20	8.38	12.28	26.64	24.43
Q5SSJ5	**HP1BP3#**	Heterochromatin protein 1-binding protein 3	2	6	13	15	3.12	10.71	25.87	27.64
Q58FF8	HSP90AB2P	Putative heat shock protein HSP 90-beta 2	6	-	-	2	8.86	-	-	3.89
P08107	**HSPA1A#**	Heat shock 70 kDa protein 1A/1B	3	-	5	5	6.23	-	10.38	9.08
P54652	**HSPA2#**	Heat shock-related 70 kDa protein 2	3	-	7	9	5.8	-	13.36	17.7
P04792	**HSPB1#**	Heat shock protein beta-1	2	2	2	2	4.25	2.77	4	4
Q9NZI8	IGF2BP1	Insulin-like growth factor 2 mRNA-binding protein 1	4	5	12	15	57	7.42	20.61	15.59
O00425	**IGF2BP3**	Insulin-like growth factor 2 mRNA-binding protein 3	3	3	9	8	6.03	5.03	17.24	15.92
Q12905	**ILF2#**	Interleukin enhancer-binding factor 2	9	-	18	27	18.29	-	26.79	40.47
Q12906	ILF3#	Interleukin enhancer-binding factor 3	23	19	48	53	42.2	35.75	60.93	74.73
Q96G21	**IMP4**	U3 small nucleolar ribonucleoprotein protein IMP4	-	3	2	4	-	6.11	2.35	7.13
Q07666	**KHDRBS1**	KH domain-containing, RNA-binding, signal transduction-associated protein 1	3	-	5	4	4.51	-	7.85	6.39
P48668	KRT6C	Keratin, type II cytoskeletal 6C	-	6	-	5	-	10.19	-	8.84
P83111	LACTB	Serine beta-lactamase-like protein LACTB, mitochondrial	2	3	2	-	3.56	4.77	2.4	-
P02545	LMNA	Lamin-A/C	3	-	-	5	4.97	-	-	9.82
Q9BXY0	**MAK16**	Protein MAK16 homolog	-	4	5	4	-	6.29	9.39	8.01
P43243	**MATR3#**	Matrin-3	4	15	22	25	7.12	29.4	36.94	40.98
Q9BYG3	**MKI67IP**	MKI67 FHA domain-interacting nucleolar phosphoprotein	7	5	6	5	12.02	7.21	11.84	7.51
O00566	**MPHOSPH10**	U3 small nucleolar ribonucleoprotein protein MPP10	-	6	4	5	-	11.27	3.88	10.01
Q9BQG0	**MYBBP1A#**	Myb-binding protein 1A	5	11	5	14	10.67	20.55	11.75	27.46
O00159	MYO1C#	Myosin-Ic	-	2	3	4	-	4.43	5.67	7.7
Q9H0A0	**NAT10#**	N-acetyltransferase 10	4	3	4	7	8.49	6.54	7.26	13.43
P19338	NCL	Nucleolin	5	-	7	9	8.15	-	12.97	18.66
Q9Y221	NIP7	60S ribosome subunit biogenesis protein NIP7 homolog	-	2	4	-	-	3.74	5.8	-
O15226	NKRF#	NF-kappa-B-repressing factor	-	8	11	12	-	12.94	22.84	22.1
Q9H8H0	**NOL11**	Nucleolar protein 11	-	9	7	6	-	15.16	12.65	12.92
Q9H6R4	**NOL6**	Nucleolar protein 6	2	5	7	8	2.27	7.92	13.17	13.42
Q9UMY1	NOL7	Nucleolar protein 7	-	5	3	3	-	8.47	6	5.82
Q9Y3C1	**NOP16**	Nucleolar protein 16	2	3	3	4	4	6.58	3.74	8.07
P46087	**NOP2**	Putative ribosomal RNA methyltransferase NOP2	11	12	22	23	19.36	25.03	37.05	40.73
O00567	**NOP56#**	Nucleolar protein 56	13	15	24	27	23.87	22.4	43.65	44.13
Q9Y2X3	**NOP58#**	Nucleolar protein 58	9	-	17	19	12.35	-	29.8	31.99
Q14980	**NUMA1**	Nuclear mitotic apparatus protein 1	6	4	9	12	11.12	8.69	17.94	23.27
Q13310	**PABPC4#**	Polyadenylate-binding protein 4	4	6	7	10	7.81	6.46	14.6	15.16
Q9NWT1	PAK1IP1	p21-activated protein kinase-interacting protein 1	3	2	2	3	6	3.47	2.92	5.89
Q15365	PCBP1#	Poly(rC)-binding protein 1	2	-	2	-	3.8	-	4.01	-
Q14690	**PDCD11**	Protein RRP5 homolog	13	19	27	28	24.61	37.82	56.56	54.56
O00541	**PES1**	Pescadillo homolog	3	4	7	5	6.72	9.09	12.5	9.42
Q96HS1	PGAM5	Serine/threonine-protein phosphatase PGAM5, mitochondrial	3	-	4	5	4.34	-	7.22	8.01
P78527	**PRKDC**	DNA-dependent protein kinase catalytic subunit	2	-	3	-	3.27	-	3.73	-
Q9UMS4	**PRPF19#**	Pre-mRNA-processing factor 19	-	4	10	10	-	8.67	16.95	18.41
Q13523	**PRPF4B**	Serine/threonine-protein kinase PRP4 homolog	2	-	8	5	3.42	-	15.43	10.27
P26599	PTBP1#	Polypyrimidine tract-binding protein 1	10	9	14	15	14.9	18.56	24.28	23.42
Q15269	**PWP2**	Periodic tryptophan protein 2 homolog	2	6	6	6	2.73	11.3	11.52	11.13
Q9P0K7	**RAI14**	Ankycorbin	7	-	-	2	11.95	-	-	4
Q9UKM9	RALY#	RNA-binding protein Raly	3	11	20	23	6.36	17.52	26.01	28.69
Q96PK6	**RBM14**	RNA-binding protein 14	-	5	7	9	-	8.46	13.26	17.49
Q96T37	**RBM15**	Putative RNA-binding protein 15	3	-	6	8	3.57	-	12.18	15.66
P49756	**RBM25**	RNA-binding protein 25	2	-	-	5	3.6	-	-	9.28
Q9NW13	**RBM28**	RNA-binding protein 28	2	6	10	8	4.01	12.02	18.29	14.38
P42696	**RBM34**	RNA-binding protein 34	-	4	2	3	-	8	4.45	6.33
Q9BWF3	**RBM4**	RNA-binding protein 4	-	2	4	3	-	4.22	7.82	4.92
P38159	RBMX#	Heterogeneous nuclear ribonucleoprotein G		5	15	12	-	7.4	25.26	21.86
O95758	**ROD1**	Regulator of differentiation 1	-	2	5	4	-	4.42	8.43	6.59
Q9H9Y2	**RPF1**	Ribosome production factor 1	-	4	2	3	-	7.3	3.59	6
Q9H7B2	**RPF2**	Ribosome production factor 2 homolog	3	7	8	8	6.15	12.33	13.61	14
P62913	RPL11#	60S ribosomal protein L11	2	-	2	4	3.16	-	4	7.32
P30050	RPL12	60S ribosomal protein L12	2	-	2	2	4	-	4	2.79
P26373	RPL13#	60S ribosomal protein L13	2	-	-	4	3.46	-	-	8.4
P50914	RPL14#	60S ribosomal protein L14	2	-	2	3	3.59	-	4.01	3.06
P61313	RPL15#	60S ribosomal protein L15	2	-	3	3	4.03	-	4.97	6.04
P62829	RPL23#	60S ribosomal protein L23	3	-	5	3	2	-	8.8	4.02
P62750	RPL23A#	60S ribosomal protein L23a	2	-	2	-	3.57	-	4.39	-
P61353	RPL27	60S ribosomal protein L27	2	-	6	5	4	-	10.64	8.68
P39023	RPL3#	60S ribosomal protein L3	6	-	9	8	9.09	-	18.72	15.38
Q9Y3U8	RPL36#	60S ribosomal protein L36	2	-	2	3	3.54	-	4.38	4.94
P46777	RPL5	60S ribosomal protein L5	-	4	3	-	-	6.66	5.48	-
Q02878	RPL6#	60S ribosomal protein L6	3	7	11	13	6.41	14.44	17.38	21.66
P18124	RPL7#	60S ribosomal protein L7	4	-	3	6	-	5.31	6.6	11.46
P62424	RPL7A#	60S ribosomal protein L7a	4	-	8	11	7.11	-	15.34	18.93
Q6DKI1	**RPL7L1**	60S ribosomal protein L7-like 1	-	3	3	4	-	6.44	5.02	8.34
P62263	RPS14#	40S ribosomal protein S14	3	-	-	2	6	-	-	2.26
P23396	RPS3#	40S ribosomal protein S3	3	-	2	4	4.51	-	2.55	7.39
P62241	RPS8#	40S ribosomal protein S8	2	-	3	4	4	-	4.82	8
Q9P2E9	RRBP1	Ribosome-binding protein 1	7	-	18	19	15.03	-	36.88	38.71
P56182	RRP1	Ribosomal RNA processing protein 1 homolog A	2	3	6	4	4.19	6.66	9.28	8.38
Q5JTH9	RRP12	RRP12-like protein	2	3	4	2	2.49	6.02	5.4	3.86
Q14684	RRP1B	Ribosomal RNA processing protein 1 homolog B	4	4	11	12	7.29	7.06	18.98	20.86
Q9Y3A4	**RRP7A**	Ribosomal RNA-processing protein 7 homolog A	-	2	-	3	-	4.13	-	4.17
O43159	**RRP8**	Ribosomal RNA-processing protein 8	-	2	4	3	-	3.32	8.02	6
O43818	RRP9	U3 small nucleolar RNA-interacting protein 2	3	4	5	4	6	7.01	11.04	8.16
Q15050	**RRS1**	Ribosome biogenesis regulatory protein homolog	4	11	7	9	7.01	18.42	9.17	17.81
O76021	**RSL1D1**	Ribosomal L1 domain-containing protein 1	9	-	20	17	17.26	-	37.44	34.24
P60903	S100A10	Protein S100-A10	3	-	-	2	6	-	-	2.49
Q14151	**SAFB2#**	Scaffold attachment factor B2	6	2	10	12	12.26	2.64	20.4	22.78
Q9H7N4	**SCAF1**	Splicing factor, arginine/serine-rich 19	-	2	3	-	-	4	5.92	-
Q13435	**SF3B2**	Splicing factor 3B subunit 2	-	3	9	8	-	3.83	17.43	15.05
Q15393	**SF3B3**	Splicing factor 3B subunit 3	3	-	7	7	5.85	-	13.05	9.58
Q08170	**SFRS4**	Splicing factor, arginine/serine-rich 4	3	-	-	2	4	-	-	3.59
Q13247	SFRS6	Splicing factor, arginine/serine-rich 6	3	-	3	3	4	-	5.48	3.59
Q16629	SFRS7	Splicing factor, arginine/serine-rich 7	2	-	3	-	3.28	-	5.57	-
Q9NWH9	**SLTM**	SAFB-like transcription modulator	3	3	11	10	5.82	3.09	20.01	18.56
O60264	**SMARCA5**	SWI/SNF-related matrix-associated actin-dependent regulator of chromatin subfamily A member 5	2	-	3	3	3.35	-	6.1	5.79
O75643	**SNRNP200**	U5 small nuclear ribonucleoprotein 200 kDa helicase	-	10	23	20	-	19.16	48.66	37.55
P08621	SNRNP70	U1 small nuclear ribonucleoprotein 70 kDa	2	-	6	7	3.25	-	11.58	13.86
P18583-5	SON#	Isoform D of Protein SON	-	5	4	3	-	9.39	8.16	6.11
Q13501	SQSTM1	Sequestosome-1	2	-	2	6	3.89	-	4.37	10
Q8IYB3	SRRM1	Serine/arginine repetitive matrix protein 1	-	2	2	4	-	2.67	4.12	7.68
O95793	STAU1#	Double-stranded RNA-binding protein Staufen homolog 1	-	2	5	8	-	3.7	9.86	16.03
O75683	**SURF6**	Surfeit locus protein 6	2	-	-	3	3.1	-	-	6.24
O60506	**SYNCRIP#**	Heterogeneous nuclear ribonucleoprotein Q	8	-	11	10	14.4	-	17.7	19.41
Q92804	**TAF15**	TATA-binding protein-associated factor 2N	2	-	-	3	3.06	-	-	4
Q13148	**TARDBP**	TAR DNA-binding protein 43	2	-	3	2	4	-	5.96	3.26
Q12788	**TBL3**	Transducin beta-like protein 3	3	8	5	9	6.01	16.72	10.19	18.46
Q13428	**TCOF1**	Treacle protein	3	-	6	7	6	-	12.4	13.24
Q9NXF1	TEX10	Testis-expressed sequence 10 protein	-	4	3	3	-	6.75	4.79	6
P42166	TMPO	Lamina-associated polypeptide 2, isoform alpha	2	-	-	6	4	-	-	12.22
Q13595	**TRA2A**	Transformer-2 protein homolog alpha	2	-	5	6	2.04	-	8.55	9.86
Q14258	TRIM25#	E3 ubiquitin/ISG15 ligase TRIM25	2	-	2	2	2.66	-	2.62	4
Q14669	**TRIP12**	Probable E3 ubiquitin-protein ligase TRIP12	-	2	10	7	-	4.39	21.08	12.7
Q9BVJ6	**UTP14A**	U3 small nucleolar RNA-associated protein 14 homolog A	-	3	6	5	-	6.01	11.86	8.75
Q8TED0	**UTP15**	U3 small nucleolar RNA-associated protein 15 homolog	3	8	6	9	4.65	13.57	6.6	17.59
Q9Y5J1	**UTP18**	U3 small nucleolar RNA-associated protein 18 homolog	4	12	10	5	8.99	17.71	15.09	10.54
Q9NYH9	**UTP6**	U3 small nucleolar RNA-associated protein 6 homolog	3	3	7	7	6	6.4	13.09	12.81
P21796	**VDAC1**	Voltage-dependent anion-selective channel protein 1	2	-	-	2	2.46	-	-	4.01
Q9Y277	VDAC3	Voltage-dependent anion-selective channel protein 3	2	-	3	2	3.02	-	6.01	4.12
Q9GZL7	WDR12	Ribosome biogenesis protein WDR12	2	7	3	2	3.38	14.26	4.27	4.26
Q9UNX4	**WDR3**	WD repeat-containing protein 3	3	10	9	8	6	20.94	18.64	15.57
Q8NI36	**WDR36**	WD repeat-containing protein 36	-	8	12	17	-	19.87	25.61	33.36
Q15061	**WDR43**	WD repeat-containing protein 43	-	4	6	7	-	6.52	8.33	12
O15213	WDR46	WD repeat-containing protein 46	3	6	9	9	6	10.03	16.71	17.11
Q6RFH5	WDR74	WD repeat-containing protein 74	2	6	-	-	4.14	10.11	-	-
Q8IWA0	**WDR75**	WD repeat-containing protein 75	4	10	5	13	6.17	17.11	12.14	21.8
Q9H0D6	**XRN2**	5’-3’ exoribonuclease 2	2	6	14	12	2.71	12.14	28.48	23.17
Q7Z2W4	**ZC3HAV1**	Zinc finger CCCH-type antiviral protein 1	5	6	8	6	9.06	9.94	16.91	11.3
Q96KR1	**ZFR**	Zinc finger RNA-binding protein	10	20	34	33	15.16	35.58	42.02	48.56
Q5BKZ1	ZNF326	Zinc finger protein 326	3	-	5	7	6	-	8.37	10.49

* Represents the -log_10_ probability of a false positive (for example, Unused score of 2.0 corresponds to *p* = 0.01; Unused score of 3 corresponds to *p* = 0.001); ^†^ Genes depicted in bold and slightly larger font were knocked down using gene-specific siRNA and the knockdown effects on cell viability and virus production determined (Figure 3); # Protein IDs found to interact with Influenza virus NS1 in VirHostNet 2.0 database (as of May 2016). The table is sorted alphabetically by gene symbol. Bio 1–3 refers to each biological replicate, and Tech 1 and 2 refers to each technical replicate performed on biological replicate #3. Proteins were immunoprecipitated from IAV A/Puerto Rico/8/34 (PR8)-infected A549 cells.

## Supplementary Materials

The following are available online at http://www.mdpi.com/1999-4915/10/12/731/s1, Figure S1: Additional STRING pathways of NS1-interacting proteins. The additional pathways are: (A) Influenza Infection; (B) 3′-UTR-mediated translational regulation; (C) Metabolism of proteins; and (D) Ribosome. (E) Interaction network of all 183 proteins. Note that NUMA1 (indicated with red arrow) does not interact with any of the other 182 proteins identified in our assay. Figure S2: (A) A549 cell viability after each of 107 genes was knocked down for 91 h. Values normalized to non-silencing (N-Si). (B) Ratio of viral titer to cell viability for the indicated 11 KD A549 cells. Error bar represents S.E.M. from 2 independent experiments. *: *p* < 0.05; **: *p* < 0.005. Supplementary Movies: Three-dimensional movies (in AVI format) depicting Mock-infected cells, wild-type cells infected with IAV PR8 and stained for viral proteins M1 or M2, and *NUMA1*-KD cells infected with IAV PR8 and stained for viral proteins M1 or M2. Blue are nuclei, green is M1 staining and red is M2 staining.
